# Emergence of *Plasmodium falciparum* strains with artemisinin partial resistance in East Africa and the Horn of Africa: is there a need to panic?

**DOI:** 10.1186/s12936-024-04848-8

**Published:** 2024-01-25

**Authors:** Ashenafi Assefa, Abebe A. Fola, Geremew Tasew

**Affiliations:** 1 Ethiopian Public Health Institute, Addis Ababa, Ethiopia; 2 Institute of Global Health and Infectious Diseases, University of North Carolina School of Medicine, Chapel Hill, NC USA; 3 Center for Computational Molecular Biology, Brown University, Providence, RI USA

## Abstract

The emergence and spread of artemisinin partial resistance in East and Horn of Africa is alarming. However, artemisinin-based combination therapy (ACT) generally remains efficacious for the treatment of falciparum malaria. The emergence of partial artemisinin resistance does not currently meet the criteria to initiate change on treatment guidelines nor affect ACT routine procurement and distribution. It is high time for scientists and transitional researchers to be more critical and vigilant on further changes so that national programmes will be able to make informed decisions as well as remain alert and prepared for any change that may be required in the future.

## Background

Artemisinin-based combination therapy (ACT) is the main and most effective treatment option for uncomplicated *Plasmodium falciparum* malaria in Africa. Artemisinin-based combinations are widely used across malaria-endemic countries in Africa, where resistance to chloroquine (CQ) and other anti-malarials [1]. is well established. In 2000, the first ACT partial resistance cases were reported in South East Asia (SEA), which has been the epicentre of anti-malarial resistance and treatment failure emergence [2, 3]. The major artemisinin-based combinations currently used in Africa are artemether-lumefantrine (AL), artesunate-amodiaquine (AA), dihydroartemisinin-piperaquine (DHAPPQ), artesunate-mefloquine (AM), and most recently artesunate-pyronaridine (AP). In Ethiopia, AL has been the first-line treatment for uncomplicated *P. falciparum* and second-line for *Plasmodium vivax* malaria since 2005 and has retained high efficacy (Table 1) [4].

**Table 1:** High ACT therapeutic efficacy for uncomplicated *P. falciparum* malaria across sentinel sites in Ethiopia

Year	Study site	Drugs used	No enrolled	ACPR (%) 28 days
2007	Serbo	AL	90	97.6
2007	Wondo	AL	103	96.9
2008	Shele	AL	98	98.9
2011	Wondo	AL	90	92.5
2011	Shele	AL	100	96.7
2010	Pawe	AL	106	99
2013	Pawe	AL	101	99
2010	Selekleka	AL	102	100
2010	Halaba	AL	89	98.7
2010	Woreta	AL	89	100
2013	Woreta	AL	79	100
2014	Humera	AL	92	98.8
2014	Metema	AL	91	98.8
2014	Metehara	AL	91	98.8
2014	Werere	AL	91	100
2017/18	Pawe	AL	89	97.8
2017/18	A/minch	AL	11	100
2017/18	Pawe	DHA-PPQ	59	100
2017/18	A/minch	DHA-PPQ	9	100
2020/21	A/minch	AL	88	98.6
2020/21	S/robit	AL	14	100
2021	Metehara	AL	80	98.7
2020/21	Hamusit	DHA-PPQ	90	98
2021	Hamusit	PY-AS (Pyramax)	90	97.7
2022/23	A/minch	AL(singledose Pq)	88	96.6
2022/23	Hamusit	AL(singledose Pq)	91	97.8
2022/23	Metehara	AL(singledose Pq)	88	97.8
2022/23	Bahirdar	AL(singledose Pq)	60	98.3

Studies conducted by the Ethiopian Public Health Institute (EPHI) for the NMCP since 2005 are included

While therapeutic efficacy studies (TES) show efficacy in Ethiopia, recent parasite genomic studies reveal molecular markers of artemisinin partial resistance (ArtR) in several African countries. These markers are found alongside fixed mutations associated with resistance to other anti-malarial drugs. New studies show an upsurge in the frequency of WHO-validated/candidate mutations in the *Kelch 13* (*k13*) gene (R561H, A675V and C469Y) in several African countries (Rwanda, Tanzania and Uganda, respectively) [5–7] Recently, a new ACT partial-resistance marker *k13* R662I and interactions with *pfhrp2/3* deletion has been reported in Ethiopia [8] and Eritrea [9] Although most of these reports of genotypic resistance are not supported by phenotypic resistance determined using standard WHO TES methods, a few studies that technically deviate from the WHO protocol reported < 90% efficacy in Angola, DRC, and Burkina Faso [10].. While phenotypic evidence of treatment failure is limited, the increasing reports of validated ArtR mutations are alarming. The emergence of ArtR in Africa may herald the emergence of ACT treatment failure, as was observed in SEA [11, 12].

Artemisinin resistance is mediated by mutations in the K13 protein. ArtR is identified by several field validated drug pressure invitro assays as well as genetic engineering studies [13–15]. These mutations likely alter ubiquitination patterns and help parasites avoid accumulation of polyubiquitinated proteins. Mutations in the propeller domain of the *k13* gene are now being routinely validated as markers of artemisinin resistance through the use of CRISPR gene editing and in vitro ring stage survival assay (RSA) [14]. However, the independent emergence of multiple partial resistance markers mostly unsupported by TES results makes policy recommendations challenging.

This commentary summarizes the recent data from East Africa and the Horn of Africa (EHoA), with an emphasis in Eritrea and Ethiopia where *k13* R622I mutation and *pfhrp2/3* deletions co-occur. The main objective of this commentary is to help inform the national malaria control programme (NMCP) and policymaker decision-making.

## What do we know

### Southeast Asia (SEA)

Clinical artemisinin resistance was first demonstrated in two parallel studies conducted in Western Cambodia in the late 2000s [2, 3]. Patients were identified with continued infection for up to 7 days after artesunate monotherapy and decreased parasite clearance rates. While propeller domain initially remained effective in SEA, ArtR emergence set the stage for ACT failure owing to rapid emergence of partner drug resistance [6]. In many areas, 35–45% of patients treated with ACT failed therapy due to the combined effect of artemisinin and partner drug resistance [12].

### East and the Horn of Africa (EHoA)

Recently, ArtR has been reported across EHoA, including Rwanda [6], Uganda [7], Tanzania [5], Kenya [16]), Ethiopia [8, 17], Eritrea [9], Somalia [18], and Sudan [19, 20].

However, ACT remain efficacious in Africa (for now). Therapeutic efficacy studies show ACT efficacy > 95% in most settings, well above the WHO 90% threshold for policy change. However, there is strong evidence that WHO-validated and candidate *k13* mutations associated with ArtR are emerging in Africa and beginning to spread. This includes R561H in Rwanda, Uganda and Tanzania; C469Y and A675V in Uganda [5, 6, 21]; and R622I in Ethiopia and Eritrea (Table 2). Recent study showed high prevalence of R622I in northern Ethiopia [8]. This mutation was first reported in two small studies in the northwest part of Ethiopia (North Gondar) with increasing prevalence over 5 years, 2.4% in 2013 and 9.5% in 2017–18 [22].

**Table 2:** K13 mutations recently reported in East Africa

Mutation	Country reporting	References
R561H	Rwanda, Uganda, Tanzania	[5–7, 16, 21]
A675V	Uganda, Rwanda	[7, 16]
C469Y/F	Rwanda	[6, 16]
P411L	Rwanda	[6, 16]
R622I	Eritrea, Ethiopia, Sudan	[8, 9, 17, 19, 20]

In Ethiopia, AL has been used for uncomplicated *P. falciparum* malaria since 2005. Accordingly, several TES have been conducted per WHO requirement, mainly led by the Ethiopian Public Health Institute (EPHI) and other groups. The results have been encouraging, with an average adequate clinical and parasitological response (ACPR) of approximately 96% The studies were conducted for programmatic purposes, and the data has been utilized by the NMCP to guide decision making and disseminated in peer-reviewed journals. However, major publications by Fola et al*.* [8], and Mihreteab et al*.* [9] reported established and expanding high prevalence of ArtR mutations (R622I) in Ethiopia and Eritrea, findings supported by a recent manuscript by Emiru et al. [17] from Ethiopia. These findings are alarming and surprising local programmes as they contradict high ACT efficacy observed during TES.

## Implications

Reports of mutations associated with ArtR are relevant to the programme and require a careful response. Multiple WHO-candidate and -validated ArtR markers have been reported across EHoA (Table 3), with *k13* 622I prevalent in Ethiopia and Eritrea. Validated makers are known to improve parasite survival in vitro during RSA, as well as delayed parasite clearance in vitro or in clinical cases assessed by day 3 parasitaemia [23]. The *k13* R622I mutation currently reported in Ethiopia and Eritrea is a WHO-validated marker. Because R622I prevalence varies from place to place within these countries and sampling was not nationally representative, nationwide genomic surveillance is warranted. Sampling across different eco-epidemiological transmission zones is important to understand the distribution of ArtR markers. The Ethiopian study by Fola et al*.* [8] used clinic-based sampling and built upon EPHI’s work on *pfhrp2*/3 deletion [24]. However, the Eritrean study byMihreteab et al*.* came from three consecutive TES surveys in 2016, 2017, 2019 [9]. The results indicate that ACT efficacy is threatened. However, the WHO definition of artemisinin partial resistance requires delay in the clearance of malaria parasites from the bloodstream or in an in vitro setting after ACT challenge. Patients infected by *P. falciparum* with partial resistance ACT may harbour parasites for more than 3 days after treatment. The remaining parasites have a better chance of survival and onward transmission unless partner drugs that remain in circulation are capable of killing them [2, 3]. Killing by partner drugs with longer half-life than artemisinin derivatives may explain the high efficacy of ACT despite partial resistance; these patients treated with an artemisinin-based combinations are fully cured if the partner drug is still efficacious [23].

**Table 3:** WHO K13 validated and candidate markers for ArtR

Validated markers	Candidate markers
F446I	P441L
N458Y	G449A
C469Y	C469F
M476I	A481V
Y493H	R515K
R539T	P527H
I543T	N537I/D
P553L	G538V
R561H	V568G
P574L	
C580Y	
R622I	
A675V	

In summary, the emergence of artemisinin partial resistance in EHoA requires immediate and more expansive clinical and genomic surveillance to closely monitor the extent of resistance and determine its clinical impact.

Despite the multiple emergences of partial resistance molecular markers, ACT remain efficacious in Ethiopia and fulfill WHO requirements for the treatment of uncomplicated *P. falciparum* malaria. Therefore, the emergence of partial artemisinin resistance does not currently meet criteria for changes in routine treatment of patients with ACT or affect routine procurement and distribution of ACT. Researchers and scientists need to work together to generate deeper and wider studies that are representative and monitor closely for evidence of decreased clinical efficacy of ACT. While increasing reports of ArtR mutations are alarming, careful and conscientious decisions will allow the programme to remain alert and to prepare for future policy changes if needed.

## Take home message to national malaria control programmes: do not panic but remain vigilant!

- Recent reports of ArtR should alert the programme to the need for robust, representative phenotypic and genomic surveillance. Genomic surveillance may need to be integrated into ongoing routine TES activities.
- Alternative artemisinin-based combinations and second-line drugs need to be sought and procurement plans developed.
- Lessons from Southeast Asia need to be reviewed and used to control the spread of ArtR parasites and for mitigation.
- Currently available data is in its early stage. Recent reports should generate concern but not panic. The programme does not need revise its current treatment regimen, nor should health providers hesitate to continue routine use of ACT. However, the programme has an opportunity to be proactive against the threat of ArtR, including close monitoring and exploring options for alternative therapies should they be required in future.
- The programme and researchers must work together to enable evidence-based decision-making.
- Mapping partner drug resistance needs to be expedited, as their efficacy is a key reason why ACT remains effective in regions affected by ArtR *P. falciparum*.

## Data Availability

No datasets were generated or analysed during the current study.
